# Can we rely on the combination of serological tests and frozen sections at the time of reimplantation for two-stage exchange hip arthroplasty in patients with a “dry tap”?

**DOI:** 10.1186/s13018-019-1223-9

**Published:** 2019-06-20

**Authors:** Chi Xu, Wei Chai, Ji-Ying Chen

**Affiliations:** Department of Orthopaedic Surgery, General Hospital of People’s Liberation Army, No.28 Fuxing Road, Haidian District, Beijing, China

**Keywords:** Periprosthetic joint infection, Frozen section, Erythrocyte sedimentation rate, C-reactive protein, Two-stage exchange arthroplasty

## Abstract

**Purpose:**

The optimal timing of reimplantation of two-stage exchange arthroplasty for periprosthetic joint infection remains unknown. The purpose of the study was to (1) evaluate performance of combination of serum erythrocyte sedimentation rate (ESR), C-reactive protein (CRP), and frozen section in predicting persistent infection at the time of second-stage hip reimplantation and (2) compare accuracies of 5 and 10 polymorphonuclear neutrophils (PMNs) per high power field (HPF) as the threshold of frozen section.

**Methods:**

We retrospectively reviewed 97 two-stage exchange hip arthroplasties from 2012–2016. Persistent infection at time of reimplantation was diagnosed using the Musculoskeletal Infection Society (MSIS) criteria. Two diagnostic models were developed. Model 1 utilized ESR, CRP, and > 5 PMNs/HPF on frozen section. Model 2 utilized ESR, CRP, and > 10 PMNs/HPF. Receiver operating characteristic (ROC) curves of the two models were generated, and areas under the curves (AUCs) were compared. A set of sensitivity analysis, using the Delphi-based consensus criteria for treatment success, was conducted to verify the accuracy of our models.

**Results:**

The overall rate of infection at reimplantation was 14.4%. AUCs for models 1 and 2 were 0.709 (95% confidence interval [CI], 0.557–0.852) and 0.697 (95% CI, 0.529–0.847), respectively. Sensitivity, specificity, positive predictive value (PPV), and negative predictive value (NPV) were 57.1%, 88.0%, 44.4%, and 92.4%, respectively, in model 1 and 42.9%, 96.4%, 66.7%, and 90.9%, respectively, in model 2. Models 1 and 2 had no significant difference in predictive values (*p* = 0.821). Results remained robust in the sensitivity analysis.

**Conclusions:**

This study reveals that the combination of serum ESR, CRP, and frozen section has limited diagnostic value in predicting persistent infection at reimplantation. Additionally, no significant difference in accuracies between 5 and 10 PMNs/HPF as the threshold of frozen section were found. There is a need for timely biomarkers with higher accuracy in diagnosing infection before reimplantation.

## Introduction

The management of periprosthetic joint infection (PJI) is challenging. Two-stage exchange arthroplasty including spacer insertion followed by reimplantation of new implants remains the preferred method for treatment of chronic PJI in North America [1]. However, the treatment outcome is unacceptable in the literature [2–4]. One key reason is the lack of a “gold standard” diagnostic method indicating the infection eradiation at the time of reimplantation [5, 6].

During the second-stage procedure, the intraoperative decision of reimplantation of new prostheses or another antibiotic spacer exchange is mainly based on the combination of serological tests, aspiration analysis, and frozen section histology. Several studies have suggested that aspiration performed on hips can be complicated by a lack of synovial fluid or a “dry tap,” especially in patients with antibiotic cement spacer [7, 8]. Additionally, aspiration analysis may be not routinely obtained before second-stage reimplantation. Therefore, the intraoperative decision-making process frequently has to rely on the combination of serological tests, such as serum erythrocyte sedimentation rate (ESR) and C-reactive protein (CRP), and frozen section analysis.

Previous studies have demonstrated limited benefits of serological tests and frozen section alone in diagnosing persistent infection at the time of reimplantation [5, 6, 9, 10]. As there is no single best diagnostic test, currently, Duwelius et al. [11] and Chen et al. [12] called for studies to evaluate the diagnostic values of a combination of the available biomarkers at reimplantation to diagnose persistent infection. The 2018 Philadelphia International Consensus on PJI also suggested a combination of available diagnostic variables should be evaluated to determine the infection status of a patient before reimplantation [13]. However, to our best knowledge, there has been no data on values of the combination of those tests in predicting persistent infection. Furthermore, the threshold of frozen section is always set at 5 polymorphonuclear neutrophils (PMNs) per high power field (HPF) for the assessment of persistent infection in the literature [14], and the value of threshold set at 10 PMNs per HPF remains unknown.

The purpose of the study was to (1) evaluate performance of the combination of serum ESR, CRP, and frozen section in predicting persistent infection at the time of second-stage hip reimplantation and (2) compare the accuracy of 5 and 10 PMNs per HPF as the threshold.

## Methods

### Patients

After Institutional Review Board approval, we retrospectively reviewed 129 patients (129 hips) who met the Musculoskeletal Infection Society (MSIS) criteria for PJI [15] and underwent a two-stage exchange arthroplasty between 2012 and 2016. Patients with megaprosthesis, prior two-stage exchange arthroplasty, spacer exchange in the interim, and patients with missing critical data were excluded. Then 97 two-stage exchanges were included in the final analysis.

Serum ESR and CRP values were routinely obtained before second-stage reimplantation in our institution. ESR and CRP levels were measured by a BN™ II System (Siemens, Marburg, Germany) and Automatic Sed-rate Analyzer 20/100 (VACUETTE) SRS/100 (Greiner Bio-One GmbH, Kremsmunster, Austria), respectively. Other clinical records of these patients were manually reviewed, including age, gender, body mass index (BMI), intraoperative cultures, and histological analyses at the time of reimplantation and follow-up data.

### Surgical protocol and frozen section

An institutional standard protocol of two-stage exchange arthroplasty was performed. During the first-stage resection arthroplasty, all implanted components were removed followed by extensive debridement and irrigation. An antibiotic-loaded cement spacer, containing 6–10 g of vancomycin and 2–4 g of meropenem, was then inserted. The combination of vancomycin and meropenem in the bone cement was utilized in accordance with our institutional infection control department, which explained that more than 90% of the organisms isolated from patients with PJI in our institution were sensitive to one or both antibiotics. Following resection arthroplasty, at least 6 weeks of systemic antibiotic therapy were prescribed. The selection of antibiotic application was based on culture sensitivity reports and institutional guidelines infectious specialists’ consultation. Reimplantation was performed after 2–4 weeks of antibiotic holiday, and the clinical course presented no signs of infection. During the second stage, the antibiotic-loaded cement spacer was removed and new prostheses were reimplanted followed by re-debridement and irrigation. Prophylactic antibiotics were continued for another 5 days after reimplantation.

Intraoperative frozen sections were routinely taken at the time of reimplantation. Three to five samples of tissues for frozen sections were obtained during surgery from the periprosthetic membrane and other periprosthetic tissues in which infection was suspected. Each sample was gathered in a separate clean specimen bag and was promptly referred to the pathology department in a sterile transport to avoid cross-contamination. All samples were stained using hematoxylin and eosin (H&E) and analyzed based on Feldman et al.’s criteria [16]. Multiple sections from each sample were classified by two experienced pathologists, and the number of PMNs per HPF (×400) was determined in 5–10 separate microscopic fields. The average was calculated as the result of the frozen section.

### Definition of persistent infection

As hip aspirations were not routinely obtained prior to the second-stage reimplantation in our institution, the diagnosis of persistent infection was based on a modified MSIS criteria [8, 17]: the presence of a sinus tract communicating with the joint at surgery or two positive intraoperative periprosthetic cultures with the same organism or fulfill two of three following minor criteria including (1) an elevated erythrocyte sedimentation rate [ESR > 30 mm/h] and C-reactive protein [CRP > 10 mg/L], (2) a single positive intraoperative periprosthetic tissue culture, and (3) a positive histologic analysis of periprosthetic tissue [> 5 neutrophils per high power field]. Additionally, persistent infection before reimplantation was also diagnosed by treatment failure following reimplantation in the sensitivity analysis (details below).

### Statistical analysis

All of the statistical analyses were performed with the statistical software packages R (http://www.R-project.org, The R Foundation). Categorical variables were presented as frequencies and percentages, and continuous variables as means and standard deviation. The differences between infection and non-infection groups were compared with the use of the Mann-Whitney test for continuous variables and the Fisher’s exact test for categorical variables. Two diagnostic models for assessment of persistent infection before reimplantation were developed, with model 1 using serum ESR and CRP with the combination of frozen section > 5 PMNs as a cutoff value and model 2 using ESR and CRP with the combination of frozen section > 10 PMNs as a cutoff value. Receiver operating characteristic (ROC) curves were generated using bootstrap resampling (times = 500) to determine the predictive values of the two models. The area under the ROC curve (AUC) with 95% confidential interval (CI) was used as a measure of diagnostic accuracy. Then the two models were compared using the DeLong method [18]. The area under the ROC curve (AUC) with 95% CI was calculated. Discriminatory value of ROC curves was interpreted as excellent (AUC 0.9–1), good (0.8–0.89), fair (0.7–0.79), poor (0.6–0.69), or fail/no discriminatory capacity (0.5–0.59) [19]. A *p* value of 0.05 was considered significant.

### Sensitivity analysis

Given no “gold standard” in predicting persistent infection before reimplantation, a set of sensitivity analysis was conducted by using treatment success following reimplantation as a proxy for infection eradication at the time of reimplantation. Treatment success was defined according to the Delphi consensus criteria proposed by Diaz-Ledezma et al. [20]: (1) infection eradication characterized by a healed wound without drainage, fistula, or pain, with no recurrence of infection; (2) no occurrence of periprosthetic joint infection-related mortality (e.g., sepsis, necrotizing fasciitis); or (3) no subsequent surgical intervention for infection after reimplantation surgery.

Only 86 patients with a minimum follow-up of 1 year were included in the sensitivity analysis. Kaplan-Meier (KM) survivorship curves were generated for follow-up at 1, 2, and 5 years. Two diagnostic models for assessment of persistent infection before reimplantation were developed, with model 3 using serum ESR and CRP with the combination of frozen section > 5 PMNs as a cutoff value and model 4 using ESR and CRP with the combination of frozen section > 10 PMNs as a cutoff value. The ROC curves of model 3 and model 4 were generated and compared according to the methods above.

## Results

According to the modified MSIS criteria, the persistent infection rate was 14.4% (14/97). Patient characteristics and organism profile were shown in Tables 1 and 2. There was no significant difference in age, gender, BMI, and CRP level between infection and non-infection groups. However, patients in the infection group had higher ESR values and PMNs per HPF than those of patients in the non-infection group.

**Table 1:** Comparisons of patient characteristics between infection and non-infection groups based on the modified MSIS criteria

	Infection (*n* = 14)	Non-infection (*n* = 83)	*P* value
Age (year)	55.60 ± 11.56	54.23 ± 16.85	0.769
Gender			0.162
Female	9 (64.29%)	36 (43.37%)	
Male	5 (35.71%)	47 (56.63%)	
BMI (kg/m^2^)	25.32 ± 3.30	24.15 ± 3.49	0.244
ESR (mm/h)	28.00 ± 23.32	17.57 ± 18.30	0.042
CRP (mg/dL)	1.34 ± 1.45	1.01 ± 1.64	0.487
Frozen section			0.046
≤ 5 PMNs	6 (42.86%)	58 (69.88%)	
5–10 PMNs	3 (21.43%)	15 (18.07%)	
> 10 PMNs	5 (35.71%)	10 (12.05%)	

*ESR* erythrocyte sedimentation rate, *CRP* C-reactive protein, *PMN* polymorphonuclear neutrophil

**Table 2:** Organism profile at first-stage resection arthroplasty and second-stage reimplantation

	Resection arthroplasty	Reimplantation
*Staphylococcus aureus*	9 (9.3%)	3 (3.1%)
Coagulase negative Staphylococci	33 (34.0%)	10 (10.3%)
Methicillin-resistant organism	5 (5.2%)	2 (2.1%)
*Enterococcus* spp.	6 (6.2%)	1 (1.0%)
*Streptococcus* spp*.*	9 (9.3%)	0 (0%)
Polymicrobial organism	13 (13.4%)	2 (2.1%)
Culture negative	32 (33.0%)	77 (79.4%)

**Fig. 1:**
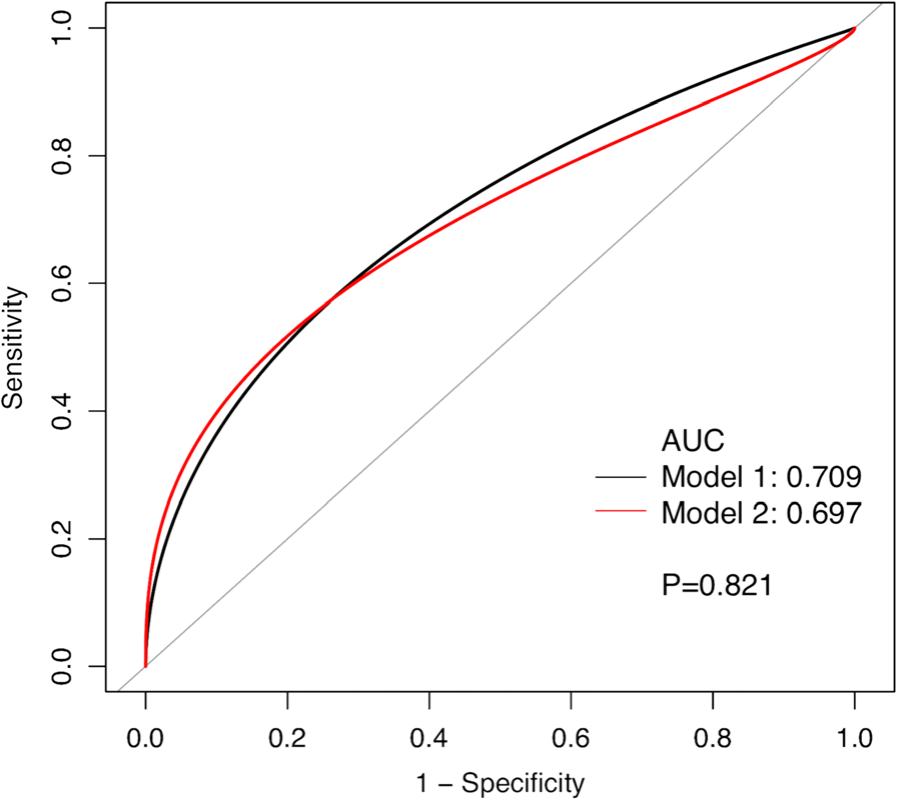
Receiver operating characteristic (ROC) curves for model 1 (ESR, CPR, and frozen section > 5 PMNs) and model 2 (ESR, CPR, and frozen section > 10 PMNs) in predicting persistent infection according to the modified MSIS criteria at the time of reimplantation

The formulas for model 1 and model 2 were presented in Table 3. Both models performed at the edge of fair, with the AUC for model 1 and model 2 at 0.709 (95% CI, 0.557–0.852) and 0.697 (95% CI, 0.529–0.847), respectively. There was no significant difference in predictive values between model 1 and 2 (*p* = 0.821, Fig. 1). With the best threshold of model 1 set at − 1.252, the sensitivity, specificity, positive predictive value (PPV), and negative predictive value (NPV) were 57.1%, 88.0%, 44.4%, and 92.4%, respectively (Table 4). With the threshold of model 2 set at − 0.644, the sensitivity, specificity, PPV, and NPV was 42.9%, 96.4%, 66.7%, and 90.9%, respectively.

**Table 3:** Formulas of the four models

Model	Formula
Diagnosis of persistent infection according to modified MSIS	
Model 1	− 2.99131 + 0.03227*ESR − 0.07385*CRP + 1.35106* (if frozen section > 5 PMNs per PHF is 1 or is 0)
Model 2	− 2.88829 + 0.03898*ESR − 0.15854*CRP + 1.81932* (if frozen section > 10 PMNs per PHF is 1 or is 0)
Diagnosis of persistent infection according to the Delphi failure (sensitivity analysis)	
Model 3	− 2.20782 + 0.01501*ESR − 0.60821*CRP + 1.08219*(if frozen section > 5 PMNs per PHF is 1 or is 0)
Model 4	− 2.02084 + 0.01980*ESR − 0.68791*CRP + 1.27583* (if frozen section > 10 PMNs per PHF is 1 or is 0)

* means multiplication

**Table 4:** Values of models in predicting persistent infection before reimplantation

	Model 1	Model 2	Model 3	Model 4
AUC	0.709	0.697	0.669	0.668
95% CI lower	0.557	0.529	0.532	0.486
95% CI upper	0.852	0.847	0.807	0.832
Best threshold	− 1.252	− 0.644	− 2.071	− 1.792
Sensitivity	57.1%	42.9%	66.7%	63.6%
Specificity	88.0%	96.4%	81.8%	81.3%
Positive predictive value	44.4%	66.7%	26.5%	33.3%
Negative predictive value	92.4%	90.9%	96.2%	93.9%

*AUC* area under ROC curve, *CI* confidential interval

### Sensitivity analysis

**Fig. 2:**
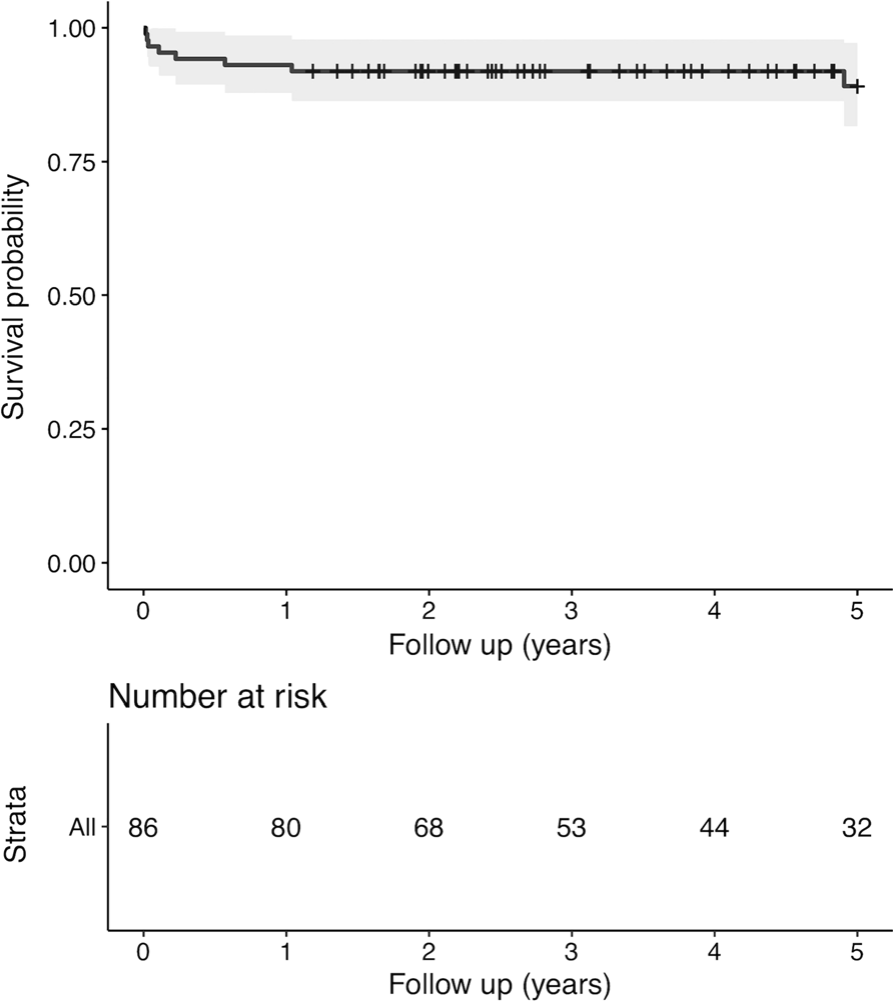
Kaplan-Meier curve of time to treatment failure according to the Delphi criteria

According to the Delphi criteria, the overall treatment success rate was 87.2% (75/86). There was no PJI-related mortality. The survivorship with treatment success as an endpoint was 93.0% (95% CI, 87.8 to 98.6%) at the 1-year follow-up, 91.9% (95% CI, 86.3 to 97.8%) at the 2-year follow-up, and 89.1% (95% CI, 81.6 to 97.2%) at the 5-year follow-up (Fig. 2).

**Fig. 3:**
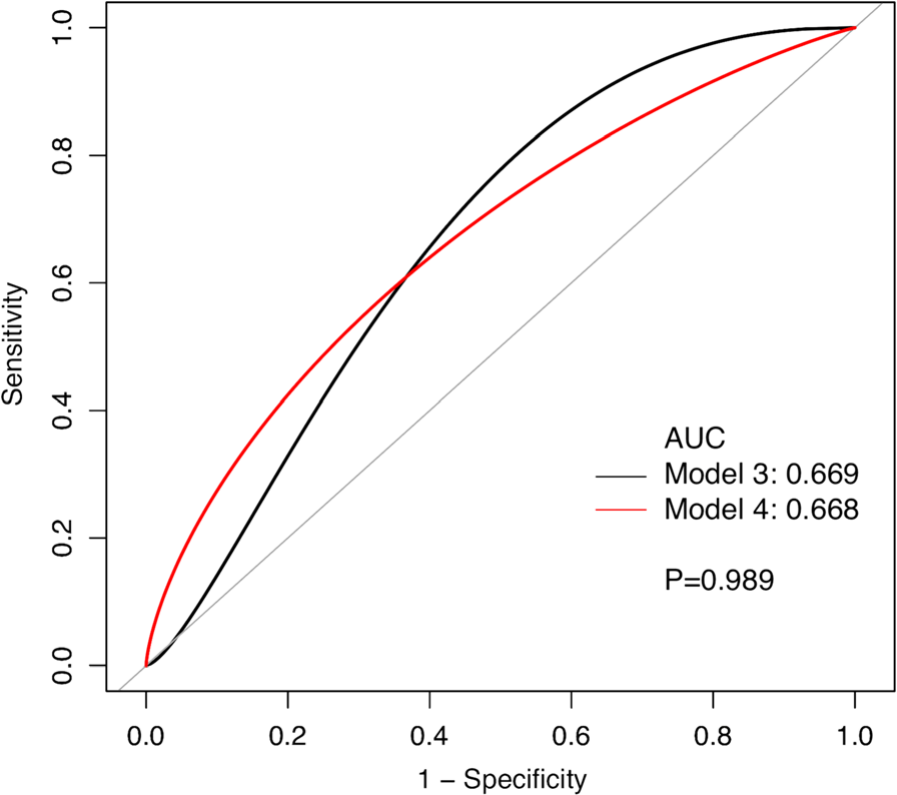
Receiver operating characteristic (ROC) curves for model 3 (ESR, CPR, and frozen section > 5 PMNs) and model 4 (ESR, CPR, and frozen section > 10 PMNs) in predicting persistent infection according to the Delphi criteria at the time of reimplantation

In the sensitivity analysis, eleven patients with treatment failure were considered as persistent infection at the time of reimplantation. The formulas for model 3 and model 4 were shown in Table 3. The results of sensitivity analysis were consistent with above results as the AUC for model 3 and model 4 was 0.669 (95% CI, 0.532–0.807) and 0.668 (95% CI, 0.486–0.832), respectively (Fig. 3). The performances were depicted in Table 4, and there was no significant difference between models 3 and 4 (*p* = 0.989).

## Discussion

It is critical to ascertain infection eradiation and the optimal timing of reimplantation. However, numerous studies have suggested no “gold test” in predicting persistent infection [5, 21–26]. The present study evaluated values of the combination of serum ESR, CRP, and frozen section in predicting persistent infection. The current results reveal limited benefits of such combination with AUCs of around 0.7 in predicting persistent infection at the time of reimplantation. Additionally, there was no significant difference between 5 and 10 PMNs as the threshold of the frozen section in predicting persistent infection.

Serum ESR and CRP are the most commonly published serological tests to screen PJI. However, these tests had low accuracies in the identification of infection eradication before reimplantation. A meta-analysis by Bian et al. reviewed eight studies on CRP and five studies on ESR and the pool data showed that the sensitivity and specificity were 45% and 73% for CRP, and 57% and 50% for ESR, respectively [25]. The rationale is that the use of systemic and local antibiotics may influence the normal inflammatory response [26]. Another reason may be that PJI with some slow-growing or less-virulent organisms (e.g., coagulase-negative staphylococcus) may result in an inapparent inflammatory response and hence may cause a significantly less evident elevation of these lab tests as compared to organisms classified as “virulent” [27, 28].

The value of the frozen section in predicting infection before reimplantation remains controversial. Previous studies suggested frozen section had high specificity but low sensitivity in the identification of persistent infection [14, 29]. Currently, a study by George et al. assessed the frozen section using MSIS criteria as a reference standard [21]. They suggested the specificity of frozen section was acceptable and higher than that of MSIS criteria despite the low sensitivity. These studies may explain all the diagnostic models of the present study have low sensitivity but an acceptable specificity.

Although the significance of synovial fluid analysis in diagnosing PJI is widely accepted [30, 31], its diagnostic value for assessment of persistent infection at second-stage reimplantation remains controversial. Zmistowski et al. reviewed 129 PJI patients undergoing two-stage exchange arthroplasty and suggested synovial white blood cell count (WBC) and synovial neutrophil percentage (PMN%) had limited benefits in diagnosing persistent infection at reimplantation, with a sensitivity of 63% and 54.5% and specificity of 62% and 60.0%, respectively [5]. A recent meta-analysis by Lee et al. showed the sensitivity and specificity of synovial WBC was only 0.37 and 0.49, respectively [32]. Furthermore, hip aspiration in patients with cement spacer is difficult due to insufficient synovial fluid [8]. Therefore, aspiration of the hip before reimplantation was not routinely obtained at my institution.

In recent years, extensive efforts have been made to identify lab tests with greater accuracies in predicting persistent infection at the time of reimplantation. Hoell et al. conducted a prospective study of 55 patients with PJI and suggested the serum interleukin 6 levels may predict persistence of infection at the time of reimplantation [6]. Several studies showed sonication of antibiotic spacers disrupted biofilm and led to higher rates of positive intraoperative cultures [33, 34]. A study by Kheir et al. indicated that a positive leukocyte esterase (LE) strip test (2+) might be indicative of persistence of infection and resulted in a higher rate of subsequent failure [22]. However, these studies were limited due to the small sample size and different definitions of persistent infection used. Additionally, these measurements were not available in all institutions. Further studies with larger sample size are required to validate these results.

There are several limitations to the present study. Most notably, there is no existing “gold standard” for the diagnosis of persistent infection. In the literature, the MSIS criteria and treatment failure following two-stage exchange arthroplasty were the most commonly used definition of persistent infection at the time of reimplantation. Therefore, the current study conducts two sets of statistical analyses by using different definitions to minimize bias. Second, the study is retrospective design and was subject to the inherent limitations. Third, we did not include aspiration analysis (e.g., synovial WBC and PMN%) in the diagnostic model as aspiration of the hip before reimplantation is not routinely performed at my institution. Lastly, it is a single-institution study, as a result, has limited external validity. However, bootstrap resampling, an internal validation method, is performed in the present study to enlarge simple sizes statistically.

## Conclusions

This study reveals that the value of the combination of serum ESR, CRP, and frozen section in the identification of persistent infection at the time of reimplantation is limited despite an acceptable specificity. Additionally, there was no significant difference for accuracies between 5 and 10 PMNs as the threshold of the frozen section. There is an urgent need for timely biomarkers with higher accuracies in predicting persistent infection at the time of reimplantation.

## Data Availability

We do not wish to share our data, because some of the patient’s data regarding individual privacy, and according to the policy of our hospital, the data could not be shared with others without permission.

## Abbreviations

AUC: Areas under the curves
CRP: C-reactive protein
ESR: Erythrocyte sedimentation rate
H&E: Hematoxylin and eosin
HPF: High power field
MSIS: Musculoskeletal Infection Society
NPV: Negative predictive value
PJI: Periprosthetic joint infection
PMN: Polymorphonuclear neutrophils
PPV: Positive predictive value
ROC: Receiver operating characteristic curves
